# Nuclear Import of a Secreted “*Candidatus* Liberibacter asiaticus” Protein is Temperature Dependent and Contributes to Pathogenicity in *Nicotiana benthamiana*

**DOI:** 10.3389/fmicb.2019.01684

**Published:** 2019-07-24

**Authors:** Xuelu Liu, Yanyan Fan, Chao Zhang, Meixue Dai, Xuefeng Wang, Weimin Li

**Affiliations:** ^1^Citrus Research Institute, Southwest University, Chongqing, China; ^2^Biotechnology Research Institute, Chinese Academy of Agricultural Sciences, Beijing, China; ^3^College of Life Science, Shandong Normal University, Jinan, China

**Keywords:** citrus HLB, secreted protein, virulence, temperature, nuclear import

## Abstract

“*Candidatus* Liberibacter asiaticus” (CLas), one of the causal agents of citrus Huanglongbing (HLB), secretes proteins with functions that are largely unknown. In this study, we demonstrated that CLIBASIA_00460, one of the CLas-encoded Sec-dependent presecretory proteins, might contribute to the phytopathogenicity of CLas. *CLIBASIA_00460* was conserved in CLas strains and expressed at a significantly higher level in citrus than in Asian citrus psyllid. *Agrobacteria*-mediated transient expression in *Nicotiana benthamiana* epidermal cells showed that the mature CLIBASIA_00460 (m460) without the putative Sec-dependent signal peptide was localized in multiple cellular compartments including nucleus at 25°C, but that nuclear accumulation was greatly decreased as the temperature rose to 32°C. When overexpressed *via* a *Potato virus X* (PVX)-based expression vector in *N. benthamiana*, m460 induced no local symptoms, but tiny necrotic spots were scattered on the systemic leaves. However, NLS-m460, which contains the SV40 nuclear localization sequence (NLS) at the N-terminus to promote nuclear import of m460, caused chlorosis and necrosis in the local leaves and severe necrosis in the systemic leaves. Taken together, these data suggest that CLIBASIA_00460 represented a novel virulence factor of CLas, and that nuclear localization of this protein was temperature dependent and positively correlated with its pathogenicity *in planta*.

## Introduction

Huanglongbing (HLB), which is also known as citrus greening, is the most destructive disease of citrus worldwide (Bové, 2006; Gottwald, 2010; da Graça et al., 2016). This disease is believed to be caused by the Gram-negative α-proteobacterium *Candidatus Liberibacter*, although Koch’s postulates have not yet been fulfilled (Wang and Trivedi, 2013; Wang et al., 2017). To date, three *Ca. Liberibacter* species have been identified to be associated with HLB, “*Ca. L. asiaticus*” (CLas), “*Ca*. *L. africanus*” (CLaf), and “*Ca*. *L. americanus*” (CLam) (Jagoueix et al., 1994; Teixeira et al., 2005; Wang et al., 2017), of which, CLas is the most prevalent and virulent. In nature, CLas is transmitted among citrus plants by Asian citrus psyllid (ACP), a phloem-feeding insect. Similar to CLam and CLaf, the bacterium CLas colonized the phloem sieve elements of citrus plants, which gradually lead to disease symptoms of leaf mottling, discolored fruits and aborted seeds, and eventually, the death of the entire plant (Bové and Ayres, 2007; Gottwald, 2010; da Graça et al., 2016).

Despite the currently unculturable status of CLas, the complete genome of this organism had been elucidated through metagenomics (Duan et al., 2009), laying a foundation for functional genomics. Accordingly, the biological functions of the CLas-encoded proteins have been investigated. For example, NttA was shown to be a ATP translocase (Vahling et al., 2010), and LasA_I_ and LasA_II_ represent two novel autotransporters, the former of which targets mitochondria in *N. benthamiana* (Hao et al., 2013). LdtR functions as a master regulator of gene expression with a role in osmotic stress tolerance (Pagliai et al., 2014, 2017), and expression of four prophage late genes (*SC1_gp025*, *SC1_gp095*, *SC1_gp100,* and *SC1_gp110*) limited the host range and culturability of CLas (Fleites et al., 2014). Moreover, a recent study showed that *CLIBASIA_00255* encoded a salicylic acid (SA) hydroxylase, thereby attenuating SA accumulation and hypersensitive response (HR) in *N. tabacum* (Li et al., 2017), suggesting the pathogenesis of CLas.

Bacterial pathogens often secret proteins (also called effector) contributing to disease pathogenesis (Stavrinides et al., 2008). In contrast to many Gram-negative bacteria, which evolve Type III, type IV or type VI secretion system (T3SS, T4SS, and T6SS) to inject effector proteins into host cells (Cambronne and Roy, 2006; Shames and Finlay, 2012; Galán and Waksman, 2018), CLas lacks these systems although it has the Sec secretion machinery (Duan et al., 2009) and the capability to release at least 86 proteins (Prasad et al., 2016). However, functions of these Sec-dependent secretory proteins are largely unknown, although one of them has been shown to target chloroplasts, cause starch accumulation and cell death in *Nicotiana benthamiana* (Pitino et al., 2016, 2018), physically interact with the citrus papain-like cysteine proteases (PLCPs), a group of defense regulators, and to reduce PLCP activity (Clark et al., 2018). In addition, two nonclassically secreted proteins, SC2_gp095 and CLIBASIA_RS00445, have been identified as functional peroxidases that dramatically suppress the transcription of *RbohB*, a key gatekeeper of H_2_O_2_-mediated defense signaling in plants (Jain et al., 2015, 2018), indicating roles of the secreted proteins in suppressing host innate immunity.

Herein, we report the pathogenic role of CLIBASIA_00460 (GenBank No. ACT56680.1), a Sec-dependent secretory protein of CLas. Using a green fluorescence protein (GFP) reporter, we demonstrated that the distribution of mature CLIBASIA_00460 (hereafter referred to as m460) in *N. benthamiana* epidermal cells was greatly influenced by temperature, and high temperature severely impeded nuclear import of m460. In addition, increasing nuclear accumulation of m460 significantly exacerbated foliar chlorosis and necrosis in *N. benthamiana*, suggesting that nuclear localization of this protein determined its pathogenicity.

## Materials and Methods

### Plants, Microbial Strains, and Growth Conditions

*N. benthamiana* plants were maintained in a greenhouse at 25°C. *Escherichia coli* DH5α was grown on Luria-Bertani (LB) medium at 37°C, while *Agrobacterium tumefaciens* strains EHA105 and GV3101 were grown on YEB supplemented with 50 μg/ml rifampicin, 50 μg/ml kanamycin, and 2 μg/ml tetracycline when necessary.

### CLas-Infected Citrus and Psyllids

The healthy adult ACPs were transferred to two-year-old CLas-infected Valencia sweet orange (*Citrus sinensis*) seedlings and maintained in a greenhouse with a temperature of 25 ± 1°C. After three-week feeding, the ACPs were collected and stored at −80°C. To extract the total RNA, the midribs of 4–5 citrus leaves showing HLB symptoms or 30–40 ACPs were pooled and quickly frozen in liquid nitrogen, then ground to a powder using an autoclaved mortar and pestle, after which they were immediately extracted with TRIzol Reagent (Invitrogen, USA). The RNA concentration and purity were determined based on the ratio of the absorbance at 260 and 280 nm measured using a DS-11 spectrophotometer (DeNoVIX Inc., USA). cDNA was synthesized by reverse transcription (RT) with random primers using the PrimeScript™ RT reagent Kit with gDNA Eraser (TaKaRa, JP). Next, quantitative polymerase chain reaction (qPCR) was performed with the primers listed in Supplementary Table S1 using a Step One Plus Real-time PCR System (Thermo Fisher Scientific) and TB Green Premix EX Taq II (Tli RNaseH Plus) (TakaRa, JP). The cycling conditions were as follows: pre-denaturation for 30 s at 95°C, followed by 40 cycles of amplification (95°C for 5 s, 52°C for 30 s, and 72°C for 30 s). Three technical replicates were conducted using total RNA extracted from CLas-infected citrus and psyllid samples, along with no-template controls and no-reverse transcription controls. The *CLIBASIA_00325* (GenBank No. CP001677.5) encoding DNA gyrase subunit A (*LasgyrA*) was employed as an internal reference, and the relative expression values were calculated by the Ct method (2^−ΔΔCt^). Statistical analyses of all data were conducted using the Student’s *t*-test (SPSS 10.0).

### *In silico* Analysis of Signal Peptide of CLIBASIA_00460

The SP sequence of CLIBASIA_00460 was predicted by using SignalP version 4.1 (Petersen et al., 2011), LipoPserver 1.0 (Juncker et al., 2003), and Phobius (Käll et al., 2004, 2007) with default settings of the algorithms for Gram-negative bacteria.

### Alkaline Phosphatase (PhoA) Assay

An *E. coli phoA* gene fusion assay system was established as previously described (Hoffman and Wright, 1985). The *phoA* gene (GenBank No. NC_000913) without its native SP-encoding sequence (hereafter referred to as *mphoA*) was cloned from *E. coli* BL21 with the primers mphoA-F (with an extra *Hin*d III restriction site at the 5′-end) and phoA-R (Supplementary Table S1) and then fused with the *Nde* I/*Xho* I double-digested pET-30a(+) using an In-Fusion HD Cloning Kit (TaKaRa, JP), resulting in pET-mphoA. Using the same strategy, pET-phoA harboring the full length *phoA* gene was constructed as a positive control.

The gene encoding CLIBASIA_00460 was cloned with the primer pair 460F/460R (Supplementary Table S1) and ligated into pMD18-T (TaKaRa, JP) to generate pMD-460. PCR was then performed of pMD-460 using the primers 460SP-F/460SP-R (Supplementary Table S1) to amplify the DNA sequence of the CLIBASIA_00460 N-terminal 26 amino acids, which contained the putative SP followed by six more residues immediately downstream. The obtained PCR product was inserted into the *Nde* I/*Hind* III double-digested pET-mphoA, resulting in pET-460SP-mphoA contained an in-frame gene fusion between the *SP* and the *mphoA* gene.

The resulting constructs pET-mphoA, pET-phoA, and p460SP-mphoA were individually introduced into the component *E. coli* BL21 cells, then subjected to PhoA activity assay on indicator LB agar containing 90 μg/ml BCIP (the chromogenic PhoA substrate), 100 mM IPTG (to induce *lacUV5* promoter and *T_7_lac* promoter), and 75 mM Na_2_HPO_4_ (to block endogenous phosphatase activity). The blue transformants were considered to have PhoA activity, while white colonies indicated a lack of PhoA activity.

### Subcellular Localization of m460 in Plant Cells

The coding sequence of m460 was amplified with the primer pair m460gfp-F/m460gfp-R (Supplementary Table S1), then cloned into *Kpn* I/*Xho* I double-digested pCAMBIA1300-35S-GFP to generate pm460-GFP, which could express a C-terminal GFP fusion protein m460-GFP. Using the same strategy, the nucleotide sequences of the SV40 nuclear localization signal (NLS) PKKKRKV (Kalderon et al., 1984) and the PKI nuclear export signal (NES) ELALKLAGL (Fukuda et al., 1997) were individually fused with *m460-GFP* to generate pNLS-m460-GFP and pNES-m460-GFP. The resulting constructs were then transformed into *A. tumefaciens* EHA105, and agroinfiltration was performed on the just expanded leaves of *N. benthamiana* at the five- to six-leaf stage as previously described (Van der Hoorn et al., 2000). To investigate the temperature effect on sublocalization of m460, four *N. benthamiana* plants (~five-leaf stage) grown at 25°C were pre-grown at high (32°C) or low temperature (18°C) for 3 days before agroinfiltration, then further incubated at 32 or 18°C. The infiltrated leaves were collected at 60 h post inoculation (hpi) to visualize the GFP fluorescence using a LSM700 confocal microscope (Zeiss, Germany). The experiment was repeated twice.

### *Agrobacterium*-Mediated PVX Infection Assay and Northern Blot Analysis

To characterize the pathogenic role of m460, the DNA fragments encoding m460, NLS-m460, and NES-m460 were amplified with the corresponding primers listed in Supplementary Table S1, then inserted into *Cla* I/*Sal* I treated pGR107, a binary plant expression vector based on *Potato virus X* (PVX; Jones et al., 1999), to generate pPVX-m460, pPVX-NLSm460, and pPVX-NESm460. The constructs were transformed into *A. tumefaciens* GV3101, and then subjected to agroinfiltration on six *N. benthamiana* seedlings at the three- to four-leaf stage as previously described (Jones et al., 1999). Total RNAs (3 μg) of the infiltrated leaves (10 dpi) or systemically infected leaves (10 dpi) at a similar developmental stage were then electrophoresed through 1.2% agarose-formaldehyde gel and transferred to Hybond-N^+^ membrane (Amersham, USA). Methylene blue solution (0.04% in 0.5 M NaAc, pH 5.2) was applied to the membrane to stain rRNA as a loading control. A specific probe complementary to the PVX coat protein-encoding gene (GenBank No. NC_011620) was prepared with the DIG labeling mix (Roche, USA) and used to detect the accumulation of PVX RNAs. The intensities of the RNA bands were quantified using ImageJ (Version 1.45S). The accumulation level of each construct was quantified as the ratio of the intensity of the PVX genomic RNA (gRNA) to that of the corresponding 28S rRNA, then normalized to that of pGR107. Each construct was evaluated in at least two independent experiments.

## Results

### Transcription of *CLIBASIA_00460* Was Upregulated in Citrus Relative to the Insect Host

**Figure 1:**
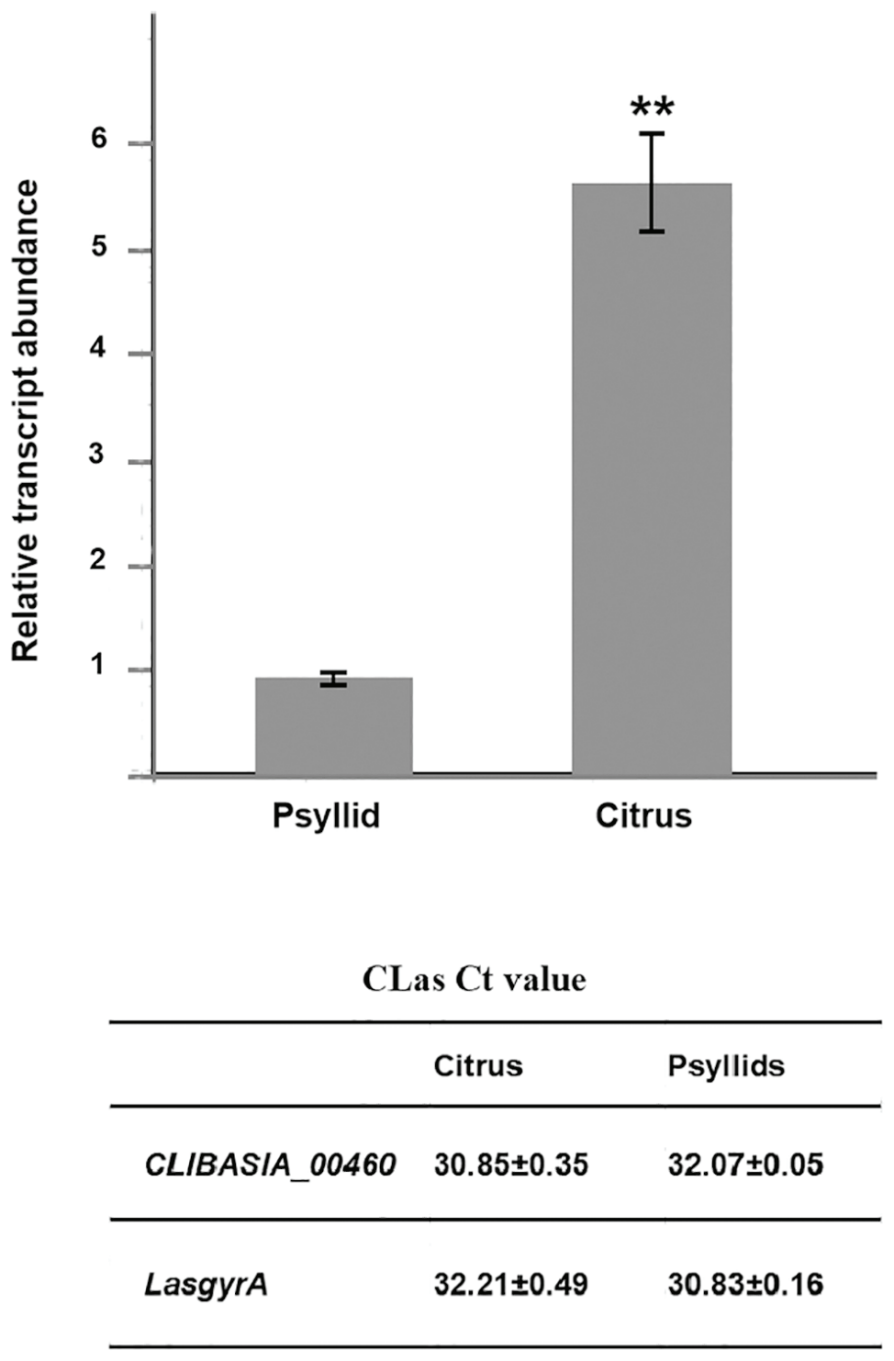
Relative expression of the *CLIBASIA_00460* gene in sweet orange and psyllids. The Ct values of *CLIBASIA_00460* and its internal standard *LasgyrA* (*CLIBASIA_00325*) are listed. Transcript abundance of *CLIBASIA_00460* was normalized against its expression in CLas-infected psyllids. Bars represent the average ± standard error of the means. The asterisks indicate a significant difference (*p* < 0.01, Student’s *t*-test).

The chromosomal gene *CLIBASIA_00460* was first annotated in CLas psy62 (Duan et al., 2009). This gene encoded a hypothetical protein and is 100% conserved among all CLas strains based on the complete genomes available to date (Supplementary Figure S1) but is absent from CLam and CLaf. To evaluate the expression levels of CLIBASIA_00460 in citrus and insect hosts, RT-qPCR was performed on total RNA extracted from CLas-infected sweet orange and ACPs. Data analysis showed that the RNA transcript of *CLIBASIA_00460* was significantly upregulated (~5.5-fold) in citrus compared to psyllids (Figure 1), implying its biological significance *in planta*.

### CLIBASIA_00460 Was a Sec-Dependent Presecretory Protein

**Figure 2:**
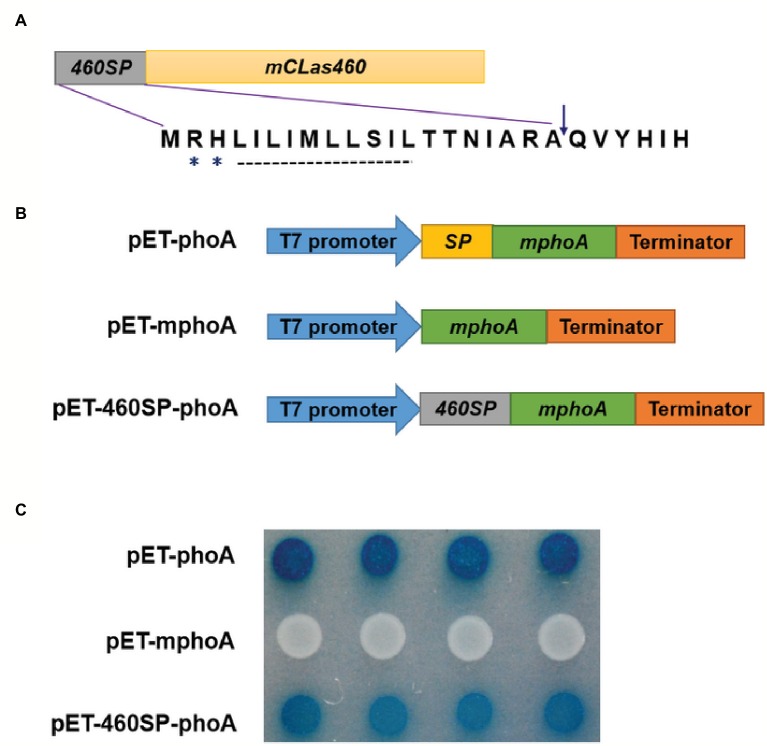
The *CLIBASIA_00460* gene encodes a Sec-dependent presecretory protein. **(A)** Gene structure of the *CLIBASIA_00460* gene. The signal peptide was named 460SP, and the mature form of CLIBASIA_00460 was m460. Two positively charged amino acids, arginine (R) and histine (H), and a central hydrophobic stretch were indicated. **(B)** Schematic of the prokaryotic expression cassettes for the *phoA* gene. **(C)** The 460SP directed the extracellular translocation of the mPhoA lacking its native SP. On indicator LB medium containing 90 μg/ml BCIP, the *E. coli* cells expressing the fusion protein 460SP-PhoA turned blue after 6 h of incubation at 37°C. IPTG (100 mM) was added to the LB medium to induce *lacUV5* promoter and *T_7_lac* promoter, which controls the *phoA* gene expression, and Na_2_HPO_4_ (75 mM) was introduced to suppress the endogenous phosphatase activity.

*In silico* analysis of CLIBASIA_00460 suggested that the protein was Sec-dependent presecretory with a putative signal peptide (namely 460SP) in the N-terminal 20 amino acids. The 460SP had two positively charged residues at the N-terminus followed by a stretch of hydrophobic amino acids (Figure 2A), sharing typical features of the Sec-dependent signal (Cranford-Smith and Huber, 2018). To validate the export of CLIBASIA_00460 *via* the Sec translocon, an *E. coli phoA* gene fusion assay was employed accordingly (Hoffman and Wright, 1985). On indicator LB agar with 90 μg/ml BCIP and 75 mM Na_2_HPO_4_, *E. coli* cells harboring pET-mphoA remained white over 24-h incubation, but those with pET-460SP-mphoA expressing the fusion protein 460SP-mphoA turned dark blue after 6 h of incubation (Figures 2B,C), indicating that 460SP successfully directed the extracellular translocation of the mPhoA moiety. Collectively, the *in silico* prediction and experimental data confirmed that CLIBASIA_00460 represented a typical Sec-dependent presecretory protein, which was in agreement with another recent report (Prasad et al., 2016).

### High Temperature Affected Subcellular Distribution of m460

**Figure 3:**
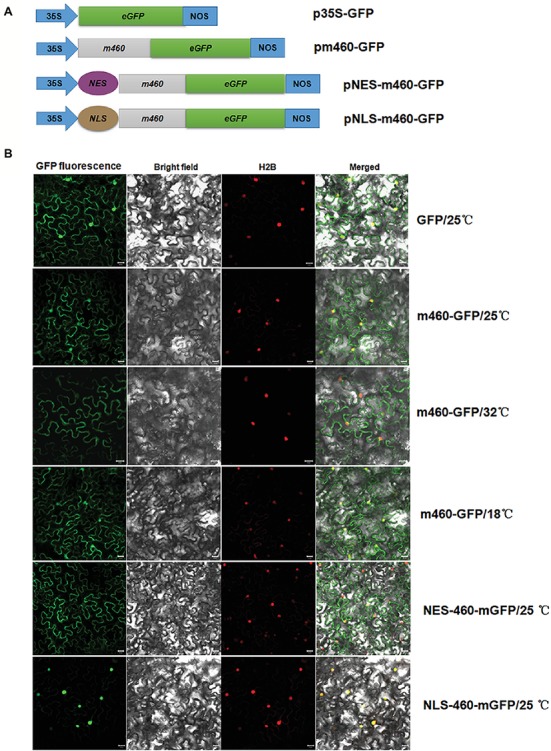
Transient expression of m460-GFP in the epidermal cells of the *N. benthamiana* leaves. **(A)** Schematic of the constructs used for agroinfiltration. The 35S represents CaMV 35S RNA promoter and NOS is the nopaline synthase terminator. NES and NLS indicate PKI nuclear export signal (ELALKLAGL) and SV40 nuclear localization signal (PKKKRKV), respectively. **(B)** Subcellular localizations of m460-GFP, NES-m460-GFP and NLS-m460-GFP. The constructs were delivered into the five-leaf stage *N. benthamiana* plants continuously grown at 25°C *via* agroinfiltration. Moreover, the plasmid bearing m460-GFP was introduced into plants pre-grown at 32 or 18°C for 3 days before agroinfiltration. The infiltrated plants were further grown at 25, 32, or 18°C for 60 h, after which the fluorescence was viewed by confocal microscopy. H2B-RFP was used as a marker for the nucleus. The sublocalization of GFP alone at 25°C was comparable to that at 32 or 18°C (data not shown). All experiments were repeated twice with similar results.

Evaluation of the Sec-dependent presecretory protein (Cranford-Smith and Huber, 2018) revealed CLIBASIA_00460 would inevitably undergo N-terminal SP cleavage and proper folding, resulting in m460, which finally traversed the bacterial outer membrane into the host cells. To visualize the subcellular localization of m460 *in planta*, the m460 coding sequence was fused with the GFP reporter gene (Figure 3A). Transient expression of m460-GFP fusion protein in *N. benthamiana* continuously grown at room temperature (25 ± 1°C) indicated that m460 was distributed in multiple cellular compartments, including the nucleus (Figure 3B). m460 showed similar sublocalization in *N. benthamiana* that was grown at 25°C, then pretreated by low-temperature exposure (18°C) for 3 days before agroinfiltration. However, only faint green fluorescence was observed in the nuclei when m460-GFP was expressed in the plants that received the high-temperature (32°C) treatment (Figure 3B). As a control, the sublocalization of GFP alone was not altered upon temperature shift. Taken together, these data showed that nuclear import of m460 was severely restricted at high temperature but not low temperature, implying that temperature influenced the nuclear distribution of m460 *in planta*.

### m460 Has a Pathogenic Role in *N. benthamiana*

**Figure 4:**
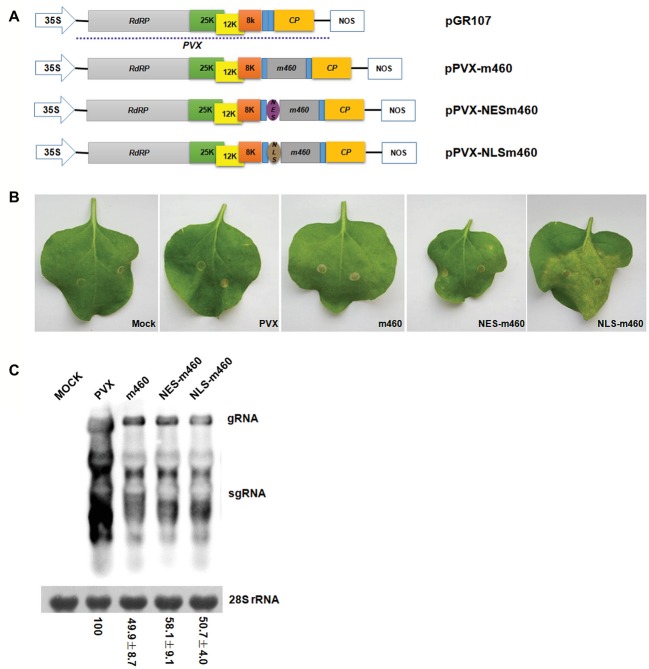
Characteristics of PVX-m460 hybrids in the infiltrated leaves of *N. benthamiana*. **(A)** Schematic diagram of the constructs based on pGR107. 35S represents CaMV 35S RNA promoter and NOS is the nopaline synthase terminator. The dashed line indicates the PVX-based viral expression vector. The open boxes represent the PVX-encoded proteins, including RNA-dependent RNA polymerase (RdRP), triple gene block movement proteins (25, 12, and 8 K), and coat protein (CP). Expression of m460, NES-m460 or NLS-m460 is driven by a duplicated CP subgenomic promoter, which is shown as a blue box. **(B)** Symptoms on leaves infiltrated with PVX, PVX-m460, PVX-NES-m460, and PVX-NLS-m460 at 10 dpi. **(C)** Northern blot analysis of total RNA from the infiltrated leaves at 10 dpi. Bands corresponding to the PVX viral RNAs are indicated, and methylene blue stain of 28S rRNA was used as a loading control. Numbers represent means with SD, indicating the relative abundance of the gRNA normalized to that of PVX. The experiments were repeated at least two times with similar results.

**Figure 5:**
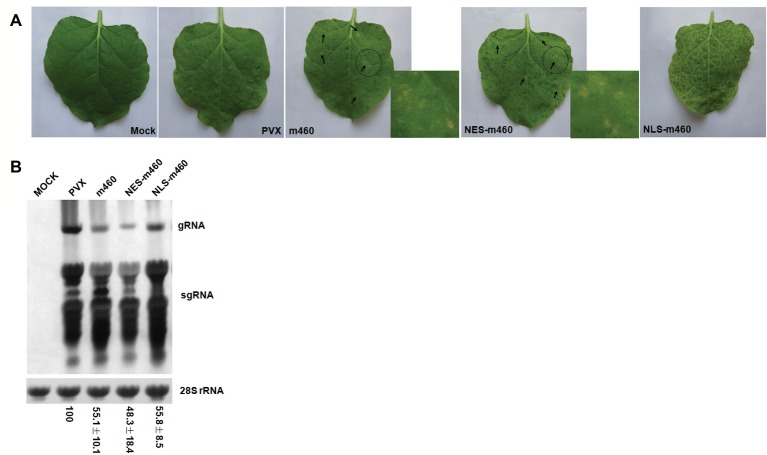
Characteristics of the PVX-m460 hybrids in systemically infected leaves of *N. benthamiana*. **(A)** Systemic symptoms of PVX, PVX-m460, PVX-NES-m460, and PVX-NLS-m460 at 10 dpi. The scattered necrotic spots induced by PVX-m460 or PVX-NES-m460 are indicated with arrows, and the regions within dashed circles were enlarged to better visualize the tiny necrosis. **(B)** Northern-blot analysis of RNA from the systemically infected leaves (10 dpi) with a similar developmental stage. Bands corresponding to the PVX viral RNAs are indicated, and methylene blue stain of 28S rRNA was used as a loading control. Numbers represent means with SD, indicating the relative abundance of the gRNA normalized to that of PVX. The experiments were repeated at least two times with similar results.

To explore the biological role of m460 in plants, the coding sequence of this protein was inserted into a PVX-based expression vector, pGR107 (Jones et al., 1999), which caused pPVX-m460 to heterologously overexpress m460 (Figure 4A), while empty pGR107 that released PVX was used as a control. Agroinfiltration assays of *N. benthamiana* showed that, similar to PVX, PVX-m460 induced no visible symptoms in the infiltrated leaves (Figure 4B), but crinkling and veinal chlorosis was observed in the systemically infected leaves at 5–6 dpi (data not shown). Continuous observations up to 10 dpi showed that the initial systemic symptoms in the PVX-infected plants gradually became chlorosis (Figure 5A). However, besides chlorosis, tiny scattered necrotic spots also developed in the systemic leaves of the plants infected with PVX-m460 (Figure 5A). Northern blot revealed that the PVX gRNAs accumulated to a higher level in both infiltrated and systemic leaves of the PVX-infected plants relative to those of the PVX-m460 infected plants at 10 dpi (Figures 4C, 5B). Collectively, the systemic symptom of tiny necrosis on the PVX-m460-infected *N. benthamiana* might not be related to the over multiplication of PVX, but instead due to heterologously expressed m460, suggesting a pathogenic role of m460 *in planta*.

### Increasing Nuclear Accumulation of m460 Promoted its Pathogenicity in *N. benthamiana*

As shown above (Figure 3B), high temperature greatly impeded nuclear import of m460 in *N. benthamiana*; therefore, further experiments were performed to evaluate the m460 subcellular redistribution. Given that the multiplication and systemic movement of PVX are decreased greatly over 30°C (Close, 1964), it is not practical to utilize the viral vector pGR107 at high temperature. To mimic the subcellular redistribution of m460 caused by temperature shifts, SV40 NLS (Kalderon et al., 1984) and PKI NES (Fukuda et al., 1997), which play the opposite roles in directing nuclear accumulation of the proteins, were individually fused with m460-GFP (Figure 3A). Transient expression analysis confirmed the expected sublocalization of NESm460 and NLSm460 in *N. benthamiana* grown at 25°C (Figure 3B).

The coding sequences of NESm460 and NLSm460 were subsequently inserted into pGR107 (Jones et al., 1999), after which the resulting PVX-NESm460 and PVX-NLSm460 (Figure 4A) infiltrated into *N. benthamiana* at 25°C. At 10 dpi, both local and systemic symptoms developed in PVX-NESm460 that were almost indistinguishable from those of PVX-m460 (Figures 4B, 5A). However, PVX-NLSm460 induced chlorosis and necrosis in the infiltrated leaves and severe interveinal necrosis in the systemic leaves (Figure 5A). Comparable amounts of the PVX gRNAs were detected in local (10 dpi) and systemic leaves (10 dpi) of *N. benthamiana* infected with PVX-m460, PVX-NLSm460, and PVX-NESm460 (Figures 4C, 5B), showing that altering the m460 nuclear import had less influence on multiplication of PVX in *N. benthamiana*. Taken together, these data indicated that the exacerbated local and systemic symptoms induced by PVX-NLSm460 were most likely because of the increased nuclear accumulation of m460, implying a positive correlation of the m460 nuclear import with its pathogenicity.

## Discussion

CLas is an intracellular bacterial pathogen with a highly reduced genome (only ~1.2 Mb) encoding 1,136 putative proteins (Duan et al., 2009), 86 of which were proposed to be secreted into host cells *via* the Sec system (Prasad et al., 2016). In this study, the pathogenic role of CLIBASIA_00460, one of the 86 Sec-dependent presecretory proteins, was demonstrated. Overexpression of m460, the mature form of CLIBASIA_00460, with a plant virus-based vector induced foliar necrosis in *N. benthamiana*, indicating a novel virulence factor of CLas *in planta*. Consistently, the transcription of *CLIBASIA_00460* was significantly upregulated in citrus relative to ACP, similar to other CLas virulence factors defined in plants (Jain et al., 2015, 2018; Li et al., 2017; Clark et al., 2018). In particular, a recent study showed that *CLIBASIA_00460* was highly transcribed at early stage of CLas infection in multiple citrus varieties, including citron, Washington navel orange, Pomeroy trifoliate, and Carrizo (Shi et al., 2019). These data, along with our current findings, strongly suggested that m460 might act as a critical virulence factor for plant colonization.

Knowledge of the subcellular localization of proteins may provide insight into their function. One of the CLas-encoded Sec-dependent secretory proteins, CLIBASIA_05315, has been shown to specifically target chloroplasts and induce cell death in *N. benthamiana* (Pitino et al., 2016). Herein, we found that m460 was localized in multiple subcellular compartments, including the nucleus, in *N. benthamiana* grown at 25°C. However, increasing nuclear accumulation of m460 significantly exacerbated necrosis in the *N. benthamiana* leaves, suggesting that the nucleus was a subcellular target in the virulence mechanism of m460. It is necessary to emphasize that the nuclear import of m460 was temperature dependent, and high temperature severely reduced the import of this protein into nuclei. Notably, m460 has been observed to be formed as long shape aggregates in cytoplasm but not nucleus (Pitino et al., 2016). The next work will be interesting to identify if there is other environment factor, just like temperature, affect the sublocalization of m460::GFP. During the past few decades, several proteins were found to be redistributed in eukaryotic cells upon temperature shift (Boyko et al., 2000; Gerber et al., 2000; Chatterjee and Fisher, 2003; Lehmann et al., 2005; Fujino et al., 2011), but no pathogen secreted proteins showing similar behavior in host cells have been identified to date. Nevertheless, these data collectively indicated that high temperature might alleviate the pathogenicity of m460 by preventing its nuclear import.

The potential effects of high temperature on m460 are reminiscent of the effects of heat treatment on citrus HLB. By this means, the HLB-associated symptoms and CLas titer can be efficiently reduced from CLas-infected citrus seedlings (Lo et al., 1981; Lo, 1983; Hoffman et al., 2013). Specifically, heat treatment not only kills CLas cells directly but also induces switching of CLas prophages from the lysogenic state to the lytic growth (Ding et al., 2018). Additionally, high temperature appears to regulate the expression of a range of citrus genes, thereby recovering the immune dysregulation and metabolic dysfunction of CLas-infected plants (Nwugo et al., 2016; Doud et al., 2017). It is known that pathogens, including bacteria, usually deliver secreted effector proteins into host cells, in which the effectors precisely target the specific subcellular compartments to manipulate the cellular processes or signaling pathways of the host, thereby facilitating pathogen survival and replication within the host (Stavrinides et al., 2008; Shames and Finlay, 2012; Lo Presti et al., 2015; Popa et al., 2016; Wang and Wang, 2018). CLas has an array of secreted proteins including Sec-dependent secreted protein and nonclassically secreted protein, some of which have been proposed to promote CLas infection by combating host innate immunity (Jain et al., 2015, 2018; Clark et al., 2018). The current findings regarding m460 combined with these previous reports suggest that, in addition to the addressed mechanisms of the effects of heat treatment on citrus HLB (Nwugo et al., 2016; Doud et al., 2017; Ding et al., 2018), high temperature might cause mislocalization of the CLas secreted proteins in host cells, resulting in interference with their potential roles and impeded CLas parasitism in plants. Given that proper subcellular localization is essential to ensure that effectors carry out their functions (Popa et al., 2016), discovering how high temperature disturbs the “correct” sublocalization of CLas secreted proteins in plant cells, which might lead to new ways of engineering plant disease resistance and therefore merits further investigation.

## Data Availability

The raw data supporting the conclusions of this manuscript will be made available by the authors, without undue reservation, to any qualified researcher.

## Author Contributions

WL, XW, CZ, and MD designed the experiments. XL, YF, CZ, and WL performed the experiments and analyzed the data. WL and XW wrote the manuscript.

### Conflict of Interest Statement

The authors declare that the research was conducted in the absence of any commercial or financial relationships that could be construed as a potential conflict of interest.
