# Supplementary material for: Nuclear Import of a Secreted “Candidatus Liberibacter asiaticus” Protein is Temperature Dependent and Contributes to Pathogenicity in Nicotiana benthamiana
Source: Front Microbiol. 2019 Jul 24;10:1684. doi: 10.3389/fmicb.2019.01684 (PMC6668550; doi:10.3389/fmicb.2019.01684)
Supplement: Supplementary file 1 [file Data_Sheet_1.doc]

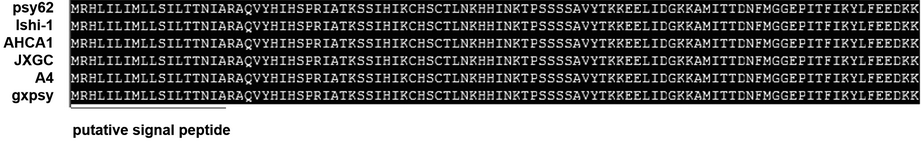


**FIGURE S1.** ClustalW alignment of the amino acid sequences of CLIBASIA_00460 from six CLas strains for which the complete genome was available, including psy62 ([NC_012985.3](https://www.ncbi.nlm.nih.gov/nuccore/NC_012985.3)), lshi-1 ([NZ_AP014595.1](https://www.ncbi.nlm.nih.gov/nuccore/NZ_AP014595.1)), AHCA1 ([NZ_CP029348.1](https://www.ncbi.nlm.nih.gov/nuccore/NZ_CP029348.1)), JXGC ([NZ_CP019958.1](https://www.ncbi.nlm.nih.gov/nuccore/NZ_CP019958.1)), A4 ([NZ_CP010804.1](https://www.ncbi.nlm.nih.gov/nuccore/NZ_CP010804.1)), and gxpsy ([NC_020549.1](https://www.ncbi.nlm.nih.gov/nuccore/NC_020549.1)). A putative signal peptide is underlined.
