# Supplementary material for: Nuclear Import of a Secreted “Candidatus Liberibacter asiaticus” Protein is Temperature Dependent and Contributes to Pathogenicity in Nicotiana benthamiana
Source: Front Microbiol. 2019 Jul 24;10:1684. doi: 10.3389/fmicb.2019.01684 (PMC6668550; doi:10.3389/fmicb.2019.01684)
Supplement: Supplementary file 2 [file Data_Sheet_2.doc]

Table S1. The primers used in this study^1^

| **Construct name** | **Primer name** | **Primer sequence (5＇-3＇)** |
| --- | --- | --- |
| pET-mphoA  pET-phoA  pMD-460  pET-460SP-mphoA  pm460-GFP  pNLS-m460-GFP  pNES-m460-GFP  pPVX-m460  pPVX-NLSm460  pPVX-NESm460  RT-qPCR | mphoA-F  phoA-R  phoA-F  phoA-R  460-F  460-R  460SP-F  460SP-R  m460gfp-F  m460gfp-R  NLSm460gfp-F  m460gfp-R  NESm460gfp-F  m460gfp-R  pvx-m460-F  pvx-m460-R  pvx-NLSm460-F  pvx-m460-R  pvx-NESm460-F  pvx-m460-R  Qgyrase296-F  Qgyrase486-R  Q460-F  Q460-R | AAGGAGATATACATATGAAACAAAGCACTATTGCACT  GTGGTGGTGGCTCGAGTTATTTCAGCCCCAGAGCGG  AAGGAGATATACATATGCGAAGCTTCCAGAAATGCCTGTTCTGGAA  --  ATGCAAGTTTATCATATCCATTC  CTATTTTTTATCTTCTTCAAATAAATA  AAGGAGATATACATATGCGTCATTTGATTTTAATA  AGGCATTTCTGGAAGCTTATGGATATGATAAACTTG  GACGAGCTCGGGTACC ATG CAAGTTTATCATATCCATTC  TGGTGTCGACTCTAGATTTTTTATCTTCTTCAAATAAATA  TGGTGTCGACTCTAGATTTTTTATCTTCTTCAAATAAATAGACGAGCTCGGGTACCATGCCTAAGAAGAAGAGAAAGGTTCAAGTTTATCATATC  --  GACGAGCTCGGGTACCATGGAGCTTGCATTAAAGCTCGCTGGTCTTGATATTAACCAAGTTTATCATATCCATTC  --  ACCAGCTAGCATCGATATGCAAGTTTATCATATCCATTC  CTTATCGGCGGTCGACCTATTTTTTATCTTCTTCAAATAAATA  ACCAGCTAGCATCGATATGCCTAAGAAGAAGAGAAAGGTTCAAGTTTATCATATC  --  ACCAGCTAGCATCGATATGGAGCTTGCATTAAAGCTCGCTGGTCTTGATATTAACCAAGTTTATCATATCCATTC  --  GTATGGCACAGGACTGGTCT  GTTAGGGCGGAAATCAACAGT  TATCATATCCATTCGCCTCGT  AGTTATAGGTTCACCTCCCAT |

1. The restriction recognition sequences of *Nde* I (yellow), *Xho* I (green), *Hin*d III (blue), *Kpn* I (pink), *Xba* I (red), *Cla* I (purple) and *Sal* I (grey) within the primers are highlighted with different color as indicated in brackets.
